# Mediastinal Lymphadenitis Caused by *Mycobacterium avium* Complex in Children: A Rare Case of Airway Compression

**DOI:** 10.1007/s00431-026-07470-9

**Published:** 2026-10-09

**Authors:** Vincent Fréret, Laureline Berteloot, Rola Abou Taam, Emmanuelle Bosdure, Harriet Corvol, Pierrick Cros, Jean-Christophe Dubus, Elsa Gachelin, Isabelle Gibertini, Eglantine Hullo, Clémence Mordacq, Nadia Nathan, Blandine Prévost, Charlotte Roy, Caroline Thumerelle, Jean-Laurent Casanova, Jérémie Rosain, Alice Hadchouel, Christophe Delacourt, Jacinta Bustamante

**Affiliations:** 1 Department of Pediatric Pulmonology and Allergology, Reference Center for Rare Lung Diseases RespiRare, Necker Hospital for Sick Children, AP-HP, Paris, France, EU; 2 Pediatric Imaging Department, Necker Hospital for Sick Children, AP-HP, Paris, France, EU; 3 INSERM UMR1163, Imagine Institute, Paris, France, EU; 4 Pediatric Pulmonology Department and Reference Center for Rare Lung Diseases RespiRare, University Timone Hospital for Children, AP-HM, Marseille, France, EU; 5 Department of Pulmonology and Reference Center for Rare Lung Diseases RespiRare, Armand Trousseau Hospital, Paris, France, EU; 6 INSERM U938, Research Center Saint-Antoine, Sorbonne University, Paris, France, EU; 7 Pediatric Department, Brest University Hospital, Brest, France, EU; 8 Aix Marseille University, C2VN, INSERM 1263, INRAE 1260, Marseille, France, EU; 9 Pediatric Department, Félix-Guyon University Hospital, Saint Denis, France, EU; 10 Pediatric Department, Tours University Hospital, Tours, France, EU; 11 Pediatric Pulmonology Unit, Couple-and-Child Hospital, Grenoble University Hospital, Grenoble, France, EU; 12 Pediatric Pulmonology Department, Lille University Hospital, Lille, France, EU; 13 Laboratory of Childhood Genetic Diseases, UMR_S933, Sorbonne University, INSERM, Armand Trousseau Hospital, Paris, France, EU; 14 Laboratory of Human Genetics of Infectious Diseases, Necker Branch, INSERM UMR1163, Imagine Institute, Necker Hospital for Sick Children, Paris, France, EU.; 15 Laboratory of Human Genetics of Infectious Diseases, University of Texas Southwestern Medical Center, Dallas, TX, USA; 16 Center for the Genetics of Host Defense, University of Texas Southwestern Medical Center, Dallas, TX, USA; 17 Howard Hughes Medical Institute, Dallas, TX, USA; 18 Department of Immunology, University of Texas Southwestern Medical Center, Dallas, TX, USA.; 19 Department of Immunology, University of Texas Southwestern Medical Center, Dallas, TX, USA; 20 Children’s Medical Center Research Institute, University of Texas Southwestern Medical Center, Dallas, TX, USA; 21 Department of Pediatrics, Necker Hospital for Sick Children, AP-HP, Paris, France, EU; 22 Université Paris Cité, Institut Imagine, Laboratory of Human Genetics of Infectious Diseases, Necker branch, INSERM UMR 1163, F-75015, Paris, France; 23 Center for the Study of Primary Immunodeficiencies, Necker Hospital for Sick Children, AP-HP, Paris, France, EU; 24 INSERM U1151, Institut Necker Enfants Malades, Université Paris Cité, Paris, France, EU

**Keywords:** *Mycobacterium avium*, Mediastinal lymphadenitis, IFN-γ, Respiratory disease, Pediatric infection

## Abstract

**Purpose.:**

Airway compression caused by intrathoracic non-tuberculous mycobacteria (NTM) lymphadenitis is particularly uncommon in otherwise healthy children and remains poorly characterized. We aim to describe the clinical, radiological, microbiological, and immunological features of a French pediatric cohort diagnosed with mediastinal *Mycobacterium avium* complex (MAC) lymphadenitis.

**Methods.:**

We conducted a retrospective multicenter study that included children diagnosed with bacteriologically confirmed intrathoracic MAC lymphadenitis in France between 2009 and 2024. Patients with HIV-related immunodeficiency or known inborn errors of immunity were excluded. Immunological investigations were performed, including the secretion of IL-12 and IFN-γ secretion by whole blood assay.

**Results.:**

Ten children with intrathoracic MAC lymphadenitis with airway compression were included. In most cases, the diagnosis required invasive sampling. *Mycobacterium avium* was identified in nine patients and *M. intracellulare* in one patient. Main respiratory manifestations were dyspnea, persistent cough, and abnormal breath sounds. Chest computed tomography (CT) scans revealed mediastinal lymphadenopathies causing airway compression, ventilation disorders, and air trapping. Following prolonged antibiotic treatment, all patients showed clinical improvement and reduction in lymph node size, although residual calcifications frequently persisted. IFN-γ secretion was impaired in all five patients who underwent specific immunological testing, consistently lower compared with healthy controls.

**Conclusion.:**

This study highlights a rare presentation of airway compression in children characterized by MAC mediastinal lymphadenitis. These findings underscore the importance of considering NTM infections in the differential diagnosis of persistent mediastinal lymphadenitis in young children, and highlight the frequent need for invasive sampling to establish microbiological diagnosis. Although no overt immunodeficiency was identified, immune evaluation, particularly exploration of the IFN-γ-mediated immunity, may be relevant in these patients.

## INTRODUCTION

Non-tuberculous mycobacteria (NTM) are opportunistic environmental acid-fast bacilli (AFB), commonly found in natural or urban water sources and in soils [1], and transmitted primarily through inhalation or ingestion (e.g., contaminated water from jacuzzi and household water systems) [2], [3]. Human-to-human transmission of NTM infection has not been demonstrated, in contrast to tuberculosis (TB). NTM infections can manifest in various forms depending on the site of infection, including cutaneous, lymph node (mainly cervical), pulmonary, osseous, articular, or disseminated disease [4]. Among the NTM species responsible for pulmonary infections, the most frequently identified are *Mycobacterium avium* complex (MAC, mainly *M. avium* and *M. intracellulare*), *M. kansasii*, *M. xenopi*, *M. malmoense*, and *M. abscessus* [5], [6]. In children, cervical lymphadenitis is the predominant manifestation of NTM infection and is mainly caused by MAC species [7], [8]. In contrast, NTM mediastinal lymphadenitis remains uncommon and is less well characterized, particularly in pediatric populations.

Unlike the chronic parenchymal pulmonary forms typically observed in patients with underlying lung diseases such as cystic fibrosis or bronchiectasis, pediatric intrathoracic NTM infection may present as isolated mediastinal lymphadenitis causing airway compression. These infections usually occur in patients with underlying immunodeficiencies, including genetic defects affecting IFN-γ-mediated immunity, such as Mendelian susceptibility to mycobacterial diseases (MSMD) [9], [10], [11], [12], [13], [14], [15], [16]. However, few reports have described otherwise healthy children presenting with mediastinal lymphadenitis and airway compression caused by NTM infection [17], [18], [19]. Data regarding the clinical presentation, radiological patterns, microbiological diagnosis, and immunological features of these children remain limited.

## MATERIALS AND METHODS

### Study design

We conducted a retrospective multicenter observational study including children aged 0 to 18 years diagnosed with mediastinal lymphadenitis caused by NTM infection in France between 2009 and 2024. Cases were identified in 2024 through a national call sent by email to heads of pediatric pulmonology departments across mainland France and Reunion Island.

Eligible patients were required to have: (i) a compatible clinical presentation with intrathoracic imaging findings suggestive of mediastinal NTM infection, and (ii) microbiological confirmation of NTM infection obtained either from an intrathoracic sample, or from an extrapulmonary site. Patients with HIV infection or inborn errors of immunity (IEIs) were excluded. Clinical, radiological, microbiological, pathological, immunological, therapeutic, and follow-up data were retrospectively collected using a standardized data collection form, either on-site, by mail, or electronically, depending on each center's preference.

All investigations, including immunological and genetic analyses, were performed as part of routine clinical care. Written informed consent for genetic testing was obtained from parents when performed. In accordance with French regulations for retrospective observational studies, patients and/or their parents received an information letter and were informed of their right to object to the use of their medical data for research purposes. The study protocol was approved by the Institutional Review Board of the French Society of Respiratory Diseases (SPLF) and was registered under the CEPRO number #2025–047.

Continuous variables are reported as medians with interquartile ranges (IQRs). Statistical analyses for figure 2, were performed using the Mann-Whitney U test, and results are presented as ns (non-significant); ** p < 0.01; **** p < 0.0001.

### Immunological evaluation and whole-blood assay for IFN-γ

Immunological investigations included lymphocyte subsets (CD3+, CD4+, CD8+, CD16+, CD19+ cells), serum immunoglobulin levels (IgA, IgM, IgG, and IgE), nitro blue tetrazolium chloride (NBT), or dihydrorhodamine (DHR) tests. Venous blood samples from healthy local and travel controls and the patient were collected in heparin-containing collection tubes. These samples were diluted 1:2 in RPMI 1640 (Gibco) supplemented with 100 IU/mL penicillin and 100 μg/mL streptomycin (Gibco). We then dispensed 1 mL of each diluted blood sample into each of five wells (1 mL/well) of a 48-well plate (Nunc). These samples were incubated for 48 hours at 37°C, under an atmosphere containing 5% CO_2_/95% air, and under various activation conditions: with medium alone, with live BCG (*M. bovis*-BCG, Pasteur substrain) at a MOI of 20 BCG cells/leukocyte, or with BCG plus recombinant (rh) IL-12 (20 ng/ml; R&D Systems), or BCG plus IFN-γ (Imukin). The supernatants were then collected and subjected to ELISA. Supernatants from whole-blood stimulation experiments were assessed for the determination of IL-12p40 (#DP400, R&D Systems), and IFN-γ (#430116, BioLegend). All experiments were performed in the same laboratory in France, using the same protocol and assay kit, and at least one healthy control was tested at the same time as the patient. This assay was performed to assess impaired response to IFN-γ/IL-12 or secretion of IFN-γ/IL-12, as previously described [20], [21], [22], [23].

## RESULTS

### Clinical features and radiological findings at diagnosis of 10 children

Among the 19 physicians contacted, responses were received from 14. Ten cases of pediatric intrathoracic NTM lymphadenitis without known immunodeficiency were identified and included in this study. Eight children were of European origin, one had a mother from North Africa (P4), and one had both parents originating from Reunion Island (P1). All patients were under the age of four, with a median age of symptom onset at 22.5 months (IQR 11–36) (Table 1). There was a slight female predominance (six girls, four boys). None of these children had received the BCG vaccination. All cases were isolated or sporadic in their corresponding family, and no history of familial TB was reported neither. No patient had other infectious diseases. All patients primarily presented with respiratory symptoms: cough, dyspnea, wheezing, or biphasic breath sound, depending on the location of the lymphadenopathy. In cases 2, 3, 5, and 10, these symptoms were initially misattributed to asthma. One patient experienced significant weight loss (P6). None of the patients had hepatosplenomegaly. One patient presented with *M. avium* cervical lymphadenitis, and another developed erythema nodosum (Table 1).

The initial radiological examination was a chest X-ray, which revealed asymmetric hyperinflation, mediastinal enlargement suggestive of lymphadenopathy, or persistent parenchymal opacity; one patient also presented with associated pleural effusion (P4). These radiographic abnormalities led to contrast-enhanced thoracic computed tomography (CT) in all patients. CT scans confirmed the presence of at least one mediastinal lymphadenopathy (> 10 mm in short-axis diameter) in all children. The most commonly involved locations were the subcarinal (90%), hilar (70%), and paratracheal (40%) regions (Figure 1 & Table S1). All enlarged lymph nodes showed central necrosis at diagnosis whereas none were calcified at presentation. Airway involvement was a prominent feature, with bronchial and/or tracheal compression associated with air trapping (90%). Parenchymal abnormalities were less frequent and consisted of consolidations (30%), interpreted as atelectasis. Although patients typically underwent further staging investigations, no cases of extra-thoracic involvement were diagnosed retrospectively (Table S1).

### Microbiological diagnosis approach

The diagnostic approach in this study is outlined as follows: nine patients (90%) required deep tissue sampling. Seven underwent a biopsy of mediastinal adenopathy (4/7 via thoracotomy, 2/7 via thoracoscopy, and 1/7 via transbronchial biopsy during bronchoscopy), while two were diagnosed through aspiration of an endobronchial granuloma during bronchoscopy (Figure S1 & Table 1). The remaining patient (P10) had intrathoracic lymphadenitis on CT in the context of previously documented *M. avium* cervical lymphadenitis. Based on the clinical presentation and imaging findings, a multidisciplinary expert meeting concluded that lower respiratory tract involvement was highly probable; the resolution of wheezing symptoms following anti-mycobacterial treatment further supported this diagnosis. Direct microbiological examinations, including acid-fast bacilli staining were negative in all tested samples. In contrast, mycobacterial cultures of the respective samples allowed species identification (Table 1). The median time to culture positivity on tissue samples was 14.5 days (IQR 9–23.75, four missing data points). BAL fluids were collected from four patients, and the culture was positive in only one (P9). Molecular biology testing was performed in few patients in our study: three 16S PCRs (two on biopsies and one on BAL), as well as one 23S PCR on a biopsy sample. Only the 16S PCR from the BAL sample was positive. Three out of eight who underwent a tuberculin skin test (TST) had a positive induration (> 5 mm) (Table 1). In contrast, interferon-gamma release assays (IGRA) were negative in all cases, including the child with a TST result of 13 mm. Regarding the identified pathogens, 90% of the patients were infected with *M. avium*, while only one patient was infected with *M. intracellulare* (Table 1).

### Treatments and outcomes

The children were treated with different regimens, although the majority received multidrug antibiotic therapy (Table S2). Drug susceptibility testing was performed and did not identify any resistance, particularly to macrolide. 20% (P4, P8) of the children benefited from an initial quadruple anti-tuberculosis therapy. Notable features of the treatments administered were as follows: a triple antibiotic therapy consisting of a macrolide (clarithromycin or azithromycin) + rifamycin (rifampicin or rifabutin) + ethambutol was used in 70% of patients (n=7). Bi-antibiotic therapy with oral macrolide (clarithromycin) + rifamycin (rifabutin) was given to 20% of patients, including one who switched from rifabutin to ethambutol. One patient had another regimen of three antibiotics: ciprofloxacin + clarithromycin + rifamycin. The median duration of antibiotic therapy was 12 months. In one case, the treatment was extended by 3 months, for a total of 15 months, due to a scarring pattern of bronchial stenosis observed on bronchoscopy (P8). One treatment was stopped at 5 months due to poor compliance, while another was discontinued after 10 months following a bronchoscopy with negative culture results (P6). Regarding antibiotic-related side effects, two treatments required modification: rifabutin was discontinued after 7 months in one patient due to neutropenia, resulting in a switch to monotherapy (P3); ethambutol was discontinued after 8 months in another patient due to suspicion of retrobulbar optic neuritis (P9), which resolved after discontinuation of the drug. Systemic corticosteroids were administered in six patients, either before or concomitantly with antimycobacterial therapy. The patients’ treatments are summarized in Table S2. Nearly all patients were symptom-free at the end of antibiotic treatment (9/10). Only one patient continued to experience a persistent spastic cough, which required the use of inhaled corticosteroids (P1). However, clinical recovery did not always align with radiological improvement. Indeed, all patients had a decrease in size of lymph nodes, but eight out of 10 retained necrotic supracentimetric lymphadenopathies with calcifications at the end of treatment (Figure 1J-K). No patient developed a severe form of the disease that required prolonged oxygen therapy or ventilatory support. The average reported follow-up in specialized centers for these children was approximately 3.5 years (ranging from 12 months to 13 years), and none of the patients experienced a second episode of NTM infection or died during the follow-up period.

### Biological and genetic findings

All children underwent first-line biological evaluation, including a complete blood count (CBC), lymphocyte phenotyping, and serum levels of immunoglobulins (IgG, A, M, and E). All these biological findings are summarized in Table 2. One patient (P1) had an elevated monocyte count, which spontaneously normalized within one month, before treatment was initiated. Lymphocyte phenotyping revealed moderate lymphopenia in five of 10 children, with 80% showing decreased T cells and 80% showing decreased B cells. Half of the patients exhibited elevated IgM levels for their age, and five patients had increased IgG levels. Total IgE levels were measured in three patients and were normal. Six of the 10 patients underwent screening for chronic granulomatous disease (CGD) using the nitro blue tetrazolium (NBT) or dihydrorhodamine (DHR) tests, with normal results in all cases. Depending on the timing of the diagnosis, the IFN-γ-mediated immunity was further explored through tests measuring IFN-γ and IL-12p40 secretion and comparing with both local and travel controls. The secretion of IFN-γ has been evaluated in five patients and was significantly reduced compared with local controls in response to BCG or BCG + IL-12 (Figure 2). Secretion of IL-12p40 was similar to travel controls in response to BCG and BCG + IFN-γ. Genetic investigations were performed in eight of the 10 patients with whole-exome sequencing (WES), and did not reveal any known pathogenic variants in genes associated with susceptibility to mycobacterial infections or other IEIs.

## DISCUSSION

We describe here a French cohort of 10 children with mediastinal lymphadenitis due to non-tuberculous mycobacteria. The small sample size in our study limits the analysis to a purely descriptive approach. Nonetheless, our findings suggest a homogeneous population in terms of clinical, radiological, and biological characteristics. The causative organisms in our cohort all belong to the MAC, which aligns with previous pediatric reports such as Fergie *et al*. (1991), describing nine cases [17]. The median age in our cohort was 22.5 months, consistent with the largest literature review to date (43 patients), reporting a median age at diagnosis of 24 months [19]. Respiratory symptoms were consistently the primary presenting feature. Superficial lymphadenitis due to NTM is well documented in pediatric populations and is more common than intrathoracic infections [26], [27]. However, to our knowledge, no literature reports concurrent superficial and mediastinal MAC lymphadenitis in the same patient. None of the patients had a clinical history suggestive of underlying chronic pulmonary disease, including cystic fibrosis.

The identification of the causative organism remains the main diagnostic challenge in pediatric intrathoracic NTM lymphadenitis. Retrospective recruitment through a national call may have introduced referral bias, potentially leading to an over-representation of diagnostically challenging cases. In our cohort, only one of four BAL samples yielded MAC, whereas microbiological confirmation was mostly obtained through targeted sampling of mediastinal lymph node or endobronchial granuloma (9/10 patients). These findings highlight that BAL may be insufficient when disease is predominantly lymph-node-driven, and that microbiological diagnosis may necessitate targeted tissue sampling. Although invasive, exploration of mediastinal lymphadenopathy by thoracoscopy or thoracotomy may be required for diagnosis. Thoracoscopic approaches may offer advantages in terms of postoperative recovery and pain compared with open thoracotomy [28], [29]. None of the patients in our cohort had received BCG vaccination. Although assessing the impact of changes in BCG vaccination policy on NTM disease is challenging, given that these infections are not subject to mandatory notification, studies from France and Sweden reported a markedly increase in childhood extrapulmonary NTM disease following discontinuation of universal BCG vaccination, suggesting a potential protective effect of BCG vaccination [30], [31]. Therefore, in a child presenting with a “tuberculosis-like” mediastinal lymphadenopathy, NTM infection should be considered, especially in the absence of BCG vaccination and when immunological tests are less suggestive of *M. tuberculosis* infection (e.g., TST < 10 mm or negative IGRA). Although molecular biology techniques were not frequently utilized in our study, previous studies have shown that 16S PCR may be at least as effective as culture in detecting NTM in sputum samples [32], [33]. More recently, metagenomic next-generation sequencing (mNGS) has emerged as a promising tool, allowing detection of mycobacteria from BAL samples within only 3 days [34], [35]. However, its applicability to mediastinal NTM lymphadenitis remains uncertain.

A common radiological pattern was observed, consisting of mediastinal lymphadenopathy associated with ventilation disorders, such as air trapping or consolidation. In some cases, the imaging appearance may be compatible with *M. tuberculosis* infection due to the location and compressive effect of lymph nodes [36]. In adults, calcified lymphadenitis is more frequently observed in TB than in NTM infection (43% vs. 12%) [37]. In our cohort, lymph node evolution frequently resulted in calcification. However, the long-term functional consequences of these calcified lymph nodes remain unknown.

The optimal management of pediatric intrathoracic MAC lymphadenitis remains undefined. Although the 2020 ATS/ERS/ESCMID/IDSA guidelines provide recommendations for pulmonary NTM disease in adults, they do not specifically address children presenting with mediastinal lymphadenitis. In the absence of official specific guidelines, the treatment approach includes a combined therapy of a macrolide (azithromycin or clarithromycin), ethambutol, and rifampin for at least 12 months with the goal of achieving negative sputum cultures [6]. The reasons why some patients received dual rather than triple antimicrobial therapy cannot be fully explained retrospectively and likely reflect differences in local practice over the study period, highlighting the lack of standardized treatment recommendations for this rare condition. Surgical procedures were primarily performed for diagnostic purposes, while systemic corticosteroids were considered on a case-by-case basis in patients with significant airway compression, based on the limited evidence supporting their use as adjunctive therapy in children with tuberculosis-related airway-compressive mediastinal lymphadenitis [38]. Treatment duration cannot be guided by sputum culture conversion in this setting and should instead be individualized according to clinical presentation, radiological evolution, microbiological findings, drug susceptibility testing, and treatment tolerance. In our series, disease evolution was then assessed clinically and radiologically at 12 months after the initiation of treatment. Clinical outcomes were favorable in all cases of the cohort. However, radiological resolution was incomplete, as residual calcified lymphadenopathy persisted in most cases. Given the early onset of disease, long-term follow-up into adulthood seems interesting to assess potential respiratory sequelae.

An underlying immunodeficiency should be systematically investigated, particularly defects in IFN-γ-mediated immunity, as well as other IEIs. The rarity of these cases and the decreased IFN-γ secretion observed in stimulated whole-blood assays in five patients from our cohort suggest a possible impairment of this pathway. Although these findings raise the possibility that a subset of these patients may have subtle defects of the IFN-γ-mediated immunity, they are insufficient to support inclusion within the spectrum of MSMD. Further investigations, including genetic analyses in all patients and standardized functional assessment of the IL-12/IFN-γ pathway, are needed to clarify the potential contribution of these immune defects to intrathoracic NTM lymphadenitis in otherwise healthy children.

This cohort illustrates an uncommon but distinctive presentation of pediatric intrathoracic MAC infection, characterized by airway-compressive mediastinal lymphadenitis and prominent respiratory symptoms. Microbiological confirmation required invasive sampling in most cases, while prolonged multidrug antibiotic therapy resulted in favorable clinical and radiological improvement in all patients. Although no overt monogenic immunodeficiency was identified, immunological assessment in a subset of patients showed reduced IFN-γ secretion, suggesting a possible role of IFN-γ-mediated immune dysregulation in susceptibility in this population. Further studies are needed to clarify the underlying immune mechanisms and long-term outcomes of these rare infections. Overall, these findings emphasize the importance of considering NTM infection in the differential diagnosis of persistent mediastinal lymphadenitis in young children, and highlight the usefulness of early invasive diagnostic procedures, as well as systematic immunological evaluation, to better characterize host susceptibility.

## Supplementary Material

Figure S1

Table S1

Table S2

## Figures and Tables

**Figure 1.**
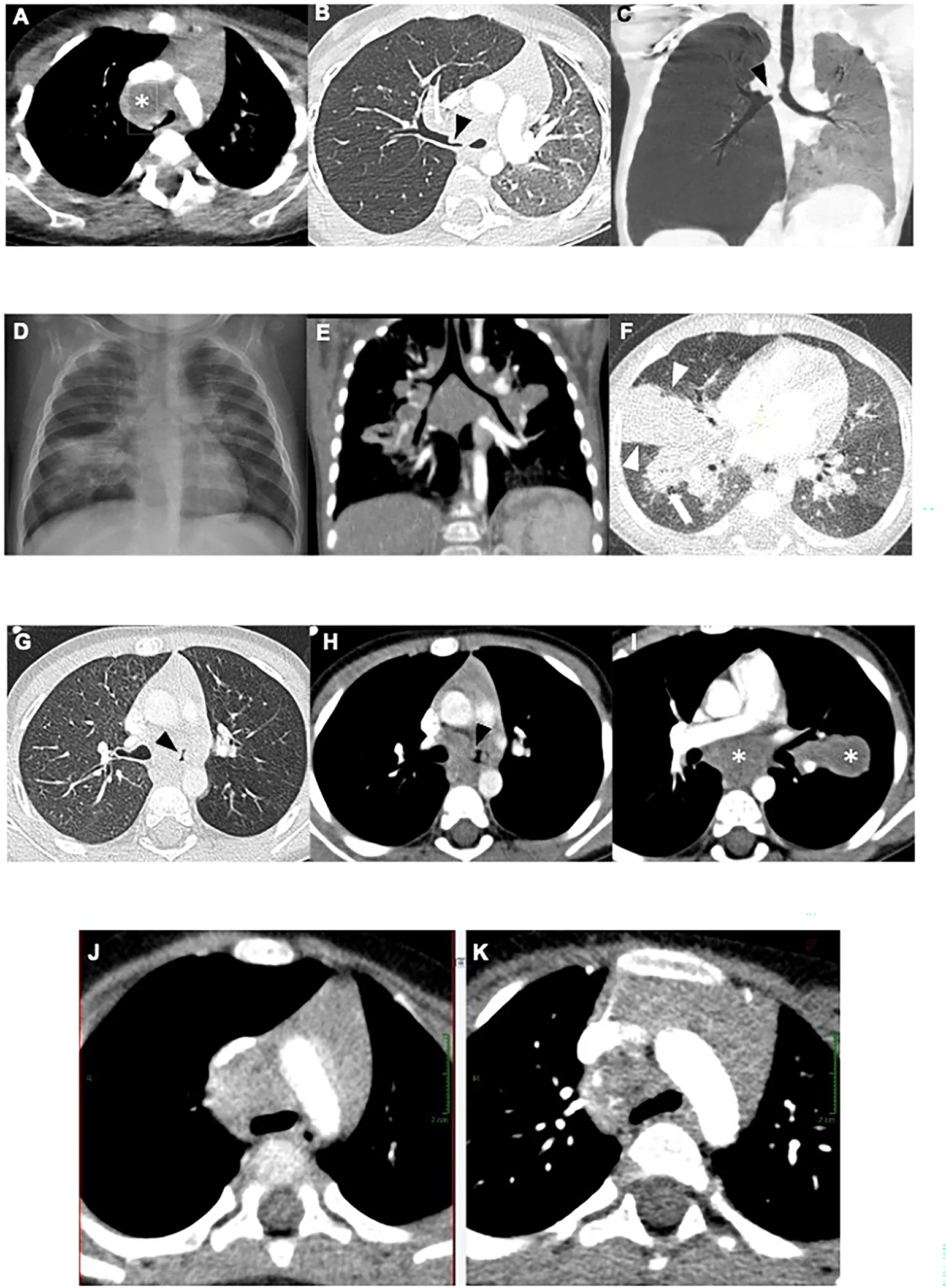
Mediastinal mycobacterial infectious diseases in pediatric patients. **A-C**. Chest CT scan at diagnosis of P2. Necrotic right paratracheal mass syndrome (asterisk), compressive on the trachea and with endobronchial extension to the right main bronchus (arrowhead). Compression and invasion of the right main bronchus were responsible for hyperinflation of the right lung, well visible on coronal Minimum Intensity Projection and mediastinal shift to the left. **D-F**. P3 chest imagery at diagnosis. **D**. Chest X-ray showed an irregularity on the right lateral wall of the trachea and the main bronchi, enlargement of the carina, and polycyclic opacities of the left hilum, raising the suspicion of compressive lymphadenopathies. Heterogeneous right opacities obscuring the right border of the heart were also present. **E** and **F**. The CT scan injection showed bilateral mediastinal and hilar confluent and lymphadenopathies. The diffuse mosaic attenuation is due to bronchial compression by lymphadenopathies. Pneumonia of the middle lobe (arrowheads) and round pneumonias (arrow) of the right lower lobe were also present. **G-I**. P7 chest CT scan at diagnosis. CT scan showed some large, confluent, partially necrotic mediastinal lymphadenopathies (medial asterisk) with significant compression and extension into the left bronchus (arrowheads). There was also a bulky necrotic left hilar adenopathy extending into the fissure (lateral asterisk). **J-K**. Evolution of P8 chest CT scan: calcified lymphadenitis. **J**. CT scan at diagnosis showed a large subcarinal lymph node. **K**. CT scan revealed a progression to calcifications of mediastinal lymphadenitis after 12 months of antibiotic treatment.

**Figure 2.**
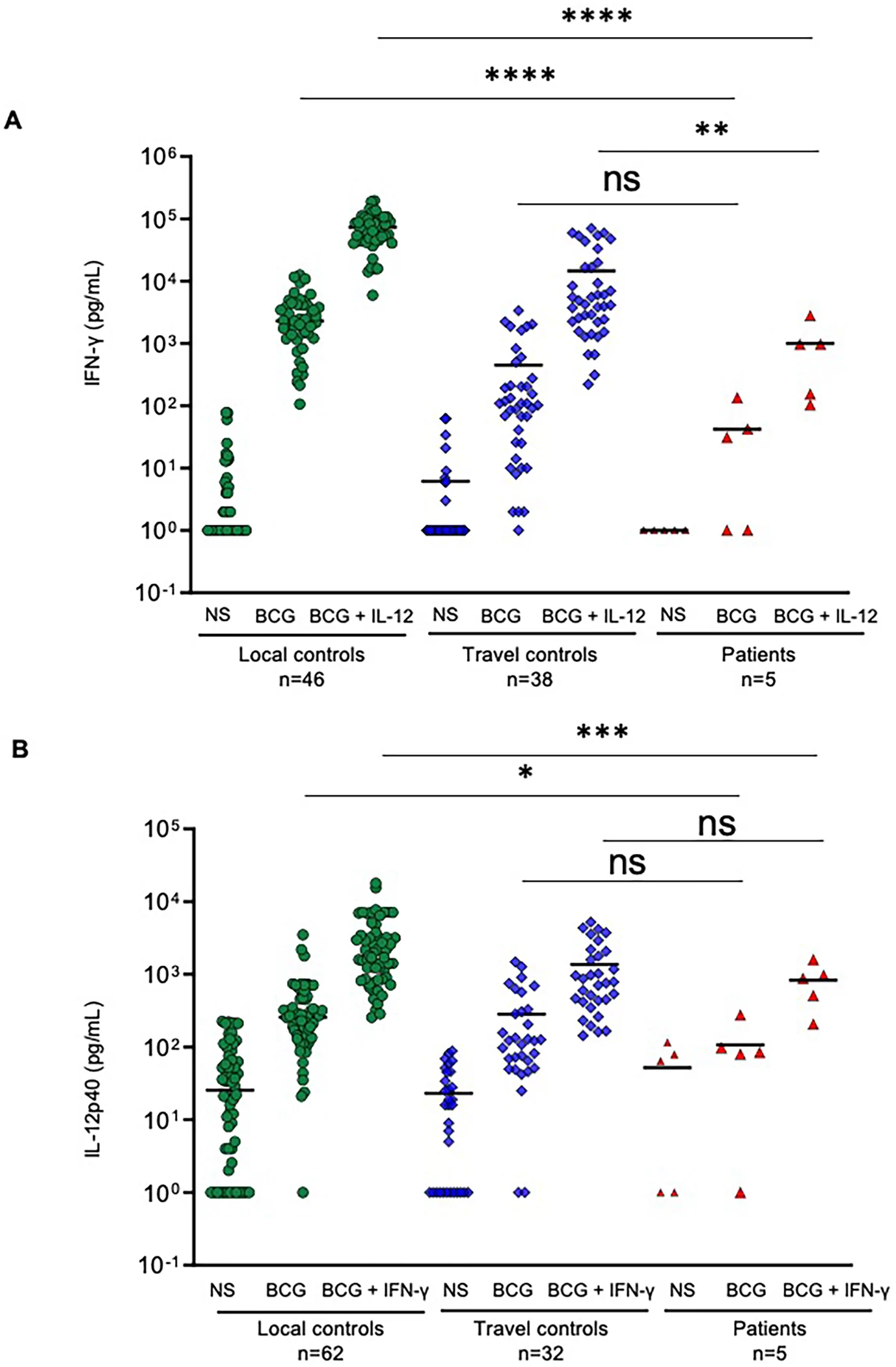
Evaluation of IFN-γ-mediated immunity by whole blood assays. Local controls refer to healthy laboratory samples, and travel controls correspond to samples collected and shipped with the patient’s specimen. **A.** Secretion of IFN-γ for the patients and healthy controls (local and travel), after activation with BCG (alone or in combination with IL-12). **B**. Secretion of IL-12p40 for the patients and healthy controls (local and travel), after activation with BCG (alone or in combination with IFN-γ).

**Table 1:** Clinical and microbiological characteristics of pediatric patients.

Cases	Age at diagnosis (months)	Symptoms and signs	Germ	Type of sample (time to positivity)	TST (mm) / IGRA
P1	9	Cough, dyspnea	*M. avium*	ADP biopsy culture (33 d)	6 / NA
P2	20	Respiratory noise, dyspnea	*M. avium*	ADP biopsy culture (12 d)	13 / Neg
P3	6	Cough, weight loss	*M. avium*	ADP biopsy culture (UK)	NA / NA
P4	36	Cough, fever	*M. avium*	ADP biopsy culture (UK)	Neg / Neg
P5	39	Cough, wheezing	*M. avium*	ADP biopsy culture (UK)	7–8 / Neg
P6	11	Respiratory noise, dyspnea, weight loss	*M. avium*	ADP biopsy culture (6 d)	Neg / NA
P7	25	Cough, fever, dyspnea	*M. avium*	Endobronchial granuloma culture (17 d)	Neg / NA
P8	11	Respiratory noise	*M. avium*	Endobronchial granuloma culture (8 d)	Neg / NA
P9	39	Cough, fever, respiratory noise, erythema nodosum	*M. intracellulare*	Biopsy ADP culture (20 d) + BAL (26 d)	Neg / Neg
P10	36	Cough, wheezing, cervical adenitis	*M. avium*	Cervical cytopuncture culture (UK)	NA / NA

ADP: adenopathy; BAL: bronchoalveolar lavage; d: days; IGRA: interferon-gamma release assay; NA: not achieved; neg: negative; N: normal; TST: tuberculin skin test; UK: unknown.

**Table 2:** Biological investigations on pediatric patients.

Cases	Leukocytes (reference ranges [24])	Lymphocyte phenotyping (reference ranges [24])	Immunoglobulins (reference ranges[25])	DHR / NBT
**P1**	Lymphocytes: 5430/μL (3320–7006), 19% Monocytes: **2800/μL** (200–1000), 9.8%, normalized one month later	CD4+: **1148/μL** (1523–3472), 32% CD8+: **323/μL** (524–1583), 9% CD19+: 1542/μL (776–2238), 43%	IgA: 0.77 g/L (0.2–0.8) IgG: **8.10 g/L** (3.3–6.2) IgM: **1.80 g/L** (0.5–1.3) IgE: 5 UI/mL (1–15)	N (DHR)
**P2**	Lymphocytes: 4740/μL (3873–6141), 58.4% Monocytes: 680/μL (300–1000), 8.4%	CD4+: 2504/μL (1573–2949), 54.8% CD8+: 900/μL (656–1432), 19.7% CD19+: 868/μL (733–1338), 19%	IgA: 0.32 g/L (0.2–0.8) IgG: 4.68 g/L (3.3–6.2) IgM: **0.46 g/L** (0.5–1.3) IgE: NR	N (DHR)
**P3**	Lymphocytes: 4700/μL (3320–7006), 44.1% Monocytes: 1000/μL (200–1000), 9.7%	CD4+: 1782/μL (1523–3472), 54% CD8+: **462/μL** (524–1583), 14% CD19+: **693/μL** (776–2238), 21%	IgA: **1.26 g/L** (0.2–0.6) IgG: **8.6 g/L** (2.3–4.4) IgM**: 0.95 g/L** (0.3–0.9) IgE: 4 UI/mL	N (NBT)
**P4**	Lymphocytes: **2000/μL** (2340–5028), 28% Monocytes: 500/μL (200–1000), 6%	CD4+: **345/μL** (870–2144), 23% CD8+: **420/μL** (472–1107), 28% CD19+: **180/μL** (434–1274), 17%	IgA: 0.4 g/L (0.3–1.2) IgG: 5.6 g/L (4.8–8.9) IgM: 0.84 g/L (0.5–1.5) IgE: NR	NR
**P5**	Lymphocytes: 5400/μL (2340–5028), 57.5% Monocytes: 830/μL (200–2000), 8.9%	CD4+: 1348/μL (870–2144), 34.3% CD8+: 1104/μL (472–1107), 28.1% CD19+: 1010/μL (434–1274), 25.7%	IgA: **1.36 g/L** (0.3–1.2) IgG: 6.45 g/L (4.8–8.9) IgM: **1.84 g/L** (0.5–1.5) IgE: NR	NR
**P6**	Lymphocytes N Monocytes N	CD4+: N CD8+: N CD19+: N	IgA: N IgG: N IgM: N IgE: NR	NR
**P7**	Lymphocytes: 3370/μL (2340–5028), 47.4% Monocytes: 670/μL (450–850), 9.4%	CD4+: **518/μL** (870–2144), 37% CD8+: **308/μL** (472–1107), 22% CD19+: **336/μL** (434–1274), 24%	IgA: 0.78 g/L (0.3–1.2) IgG: **10.15 g/L** (4.8–8.9) IgM: 1.07 g/L (0.5–1.5) IgE: NR	NR
**P8**	Lymphocytes: 4130/μL (3320–7006), 34.7% Monocytes: 730/μL (100–1000), 6.1%	CD4+: 2550/μL (1523–3472), 47% CD8+: 1520/μL (524–1583), 28% CD19+: 980/μL (776–2238), 18%	IgA: 0.54 g/L (0.2–0.8) IgG: 6.17 g/L (3.3–6.2) IgM: **1.67 g/L** (0.5–1.3) IgE: NR	N (NBT)
**P9**	Lymphocytes: N Monocytes: N	CD4+: 1540/μL (870–2144) CD8+: 1100/μL (472–1107) CD19+: **400/μL** (434–1274)	IgA: **1.35 g/L** (0.3–1.2) IgG: **10.1 g/L** (4.8–8.9) IgM: 0.64 g/L (0.5–1.5) IgE: NR	N (DHR)
**P10**	Lymphocytes: 3940/μL (2340–5028), 61.1% Monocytes: 470/μL (100–1000), 7.3%	CD4+: 1180/μL (870–2144), 31% CD8+: **1140/μL** (472–1107), 30% CD19+: 910/μL (434–1274), 24%	IgA: 1.12 g/L (0.3–1.2) IgG: **11.98 g/L** (4.8–8.9) IgM: **2.24 g/L** (0.5–1.5) IgE: 26 UI/mL (0–45)	N (DHR)

N: normal; NR: not realized.

## Data Availability

All data are either included in the manuscript or available upon request.

## What is Known – What is New

### What is Known:

- Mediastinal lymphadenitis caused by non-tuberculous mycobacteria (NTM) is a rare condition, mainly reported in immunocompromised patients, and often mimics tuberculosis on imaging.
- Diagnosis is challenging due to nonspecific clinical and radiological findings, and relies on microbiological confirmation.

### What is New:

- In this cohort, intrathoracic MAC infection in young children presented as airway-compressive mediastinal lymphadenitis rather than classical parenchymal pulmonary NTM disease.
- Bronchoalveolar lavage had low diagnostic yield, and most cases required targeted lymph node or endobronchial granuloma sampling.
- A subset of tested patients showed reduced IFN-γ secretion despite the absence of a known monogenic immunodeficiency.

## ABBREVIATIONS

BAL/F: Bronchoalveolar Lavage / Fluids
BCG: Bacillus Calmette-Guérin
CGD: Chronic Granulomatous Disease
CT: Computed Tomography
HIV: Human Immunodeficiency Virus
IEI: Inborn Error of Immunity
IFN: Interferon
IGRA: Interferon-Gamma Release Assays
IL: Interleukin
IQR: Interquartile Range
ISG: Interferon Stimulated Gene
M: Mycobacterium
MAC: *Mycobacterium avium* complex
NTM: Non-tuberculous Mycobacteria
MSMD: Mendelian Susceptibility to Mycobacterial Diseases
NF-κB: Nuclear factor-kappa B
NK: Natural Killer
TB: Tuberculosis
TST: Tuberculin Skin Test
