# Supplementary material for: Mediastinal Lymphadenitis Caused by Mycobacterium avium Complex in Children: A Rare Case of Airway Compression
Source: Eur J Pediatr. Author manuscript; Available in PMC 2026 Nov 5. (PMC13739185; doi:10.1007/s00431-026-07470-9)
Supplement: Supplementary file 1 [file NIHMS2216514-supplement-Table_S1.pdf]

Table S1: Radiological characteristics summary of pediatric patients at diagnosis.

Cases	Initial intra-thoracic lesions on CT	Extra-thoracic lesions
**P1**	Necrotic subcarinal and para-aortic ADP<br>Compression: L main bronchus<br>Condensation: no<br>Air trapping: yes	Infracentimetric mesenteric ADP
**P2**	Necrotic subcarinal, paratracheal and hilar ADP<br>Compression: R main bronchus and trachea<br>Condensation: no<br>Air trapping: yes	-
**P3**	Necrotic R para-tracheal, subcarinal and hilar ADP<br>Compression: bilateral bronchi<br>Condensation: yes<br>Air trapping: no<br>Others: ground-glass opacities	-
**P4**	Necrotic R para-tracheal and subcarinal ADP<br>Compression: trachea, R main bronchus<br>Condensation: yes<br>Air trapping: yes	Infracentimetric ADP with fatty centers in contact with the hepatic hilum<br>Recurrent ear infections
**P5**	Necrotic R subcarinal, hilar, pre-tracheal and paratracheal ADP<br>Compression: R main bronchus<br>Condensation: apical opacity<br>Air trapping: yes	-
**P6**	Necrotic subcarinal ADP<br>Compression: L main bronchus, R lobar inferior, ADP<br>Condensation: no<br>Air trapping: yes	-
**P7**	Necrotic subcarinal and L hilar ADP<br>Compression: L main bronchus<br>Condensation: no<br>Air trapping: yes	Recurrent ear infections
**P8**	Necrotic R hilar and subcarinal ADP<br>Compression: R main bronchus<br>Condensation: no<br>Air trapping: yes	-
**P9**	Necrotic L hilar and subcarinal ADP<br>Compression: L main bronchus, L inferior pulmonary vein stenosis<br>Condensation: no<br>Air trapping: yes	Recurrent laryngitis and ear infections
**P10**	Necrotic bilateral hilar ADP<br>Compression: no<br>Condensation: retractile condensation of the lateral segment of the middle lobe, lingula and basal pyramids	Cervical adenitis

ADP: adenopathy; L: left; R: right.
