# Supplementary material for: Mediastinal Lymphadenitis Caused by Mycobacterium avium Complex in Children: A Rare Case of Airway Compression
Source: Eur J Pediatr. Author manuscript; Available in PMC 2026 Nov 5. (PMC13739185; doi:10.1007/s00431-026-07470-9)
Supplement: Supplementary file 2 [file NIHMS2216514-supplement-Table_S2.pdf]

Table S2: Treatments summary of pediatric patients.

Cases	Treatments	Length (months)	Failure/switch of treatment
**P1**	Corticosteroids + CAM + RA + E	12	Inhaled corticosteroids for persistent cough
**P2**	Corticosteroids then CAM + RA + E	12	No
**P3**	CAM + rifabutin	12	Rifabutin stopped at 7 months (neutropenia), continuation of CAM alone
**P4**	Corticosteroids + initial quadruple anti-tuberculosis therapy + CAM<br>Then CAM + RA + E	12	No
**P5**	CIP + CAM + RA	12	No
**P6**	Corticosteroids + AMC then CAM + rifabutin then CAM + E	5	Non-compliance on rifabutin, switch to CAM at 2 months + E then stopped at 5 months
**P7**	Corticosteroids then CAM + RA + E	10	No
**P8**	Initial quadruple anti-tuberculosis for one month then CAM + RA + E	15	Persistence of necrotic ADP at 1 year with scarring of R main bronchus on bronchoscopy -> continued treatment for 3 months
**P9**	Corticosteroids + CAM + RA + E	12	E stopped at 8 months for suspected retrobulbar optic neuritis
**P10**	AZM + RA + E	12	No

ADP: adenopathy; AMC: amoxicillin + clavulanic acid; AZM: azithromycin; CAM: clarithromycin; CIP: ciprofloxacin; E: ethambutol; R: right; RA: rifampicin.
